# The Registrar-General's Return

**Published:** 1901-08-31

**Authors:** 


### The Registrar-General's Return.

The quarterly statement of the vital outgoings and incomings of the nation, in respect of births, marriages, and deaths, which is furnished to us at regular intervals under the authority of the Registrar-General, and which may usually be found in abstract in some odd corners of the daily newspapers, is often worthy of more careful consideration than, in the struggle for survivorship among items of intelligence, it is likely to receive from the majority of readers. The statement stands out conspicuous, if not alone, amongst much current printed matter, in the characteristic that, apart from errors incidental to faulty diagnosis, which may introduce an element of inaccuracy into death certificates and notification certificates, everything which it contains may be relied upon as being true. There is no Pro-Boerism at Somerset House; there is not even the less virulent but still mischievous taint of ordinary political partisanship. The establishment supplies nothing but facts and figures; and, in spite of the frequent correctness of the second Sir Robert Peel's wise and humorous maxim, that "there is nothing so misleading as facts, *except* figures," those now under consideration, we think, are quite sufficiently trustworthy to justify us in some small *excursus* in the direction of seeking to draw conclusions from them. We shall not go far enough seriously to tax either the patience or the endurance of our readers.

The second quarter of this current year 1901, the quarter, that is, which was made up of the months of April, May, and June, was remarkable for two main features—much sunshine and diminished mortality. The duration of bright sunshine recorded at the Royal Observatory was 670·3 hours, being 48·2 per cent. of the time during which the sun was above the horizon, the mean proportion in the twenty-four preceding second quarters having been only 34·9 per cent. The duration of bright sunshine was above the average in each month of the quarter, the excess being 73·1 hours in April, 58·2 hours in May, and 54·2 hours in June—185½ hours of bright sunshine in excess of the average. Imagination fails to attain any estimate of the countless millions of noxious bacteria which must have perished under that beneficent influence; but if we turn to the records of mortality, we may find some guidance as to its effects. The deaths registered in England and Wales during the quarter were in the proportion of 15·8 annually per thousand persons living, the average rate in the ten preceding second quarters having been 17·5. In round numbers, therefore, the average mortality of the quarter has been greater by nearly 14,000 persons than the toll actually levied by death during that through which we have just passed. In the mortality of infants under one year of age (one of the most delicate tests of the sanitary condition of a kingdom) the same improvement is exhibited. The proportion of such deaths to registered births was 118 per thousand, the average in the ten preceeding second quarters having been 128. In other words, 2,330 infants have survived who, on the averages of former years, might have been expected to perish. The third quarter is, of course, usually one of high infantile mortality; but the comparison just made, it must be remembered, is between the quarter now concluded and the corresponding quarters of former years.

The return affords valuable information, not only with regard to the diseases by which the occurring death rate has been occasioned, but also with regard to the prevalence of those diseases and the ratios borne by notified cases to mortality. We have lately expressed a doubt as to the correctness of Dr. Canney's hope that enteric fever might be excluded from camps and armies by the simple expedient of supplying the men with boiled water, and we had pleasure in admitting a letter from him in which he endeavoured to support his conclusion. Our main objection to it was based upon the fact that the care taken to obtain unpolluted water supplies in English towns, although it has been followed by a definite reduction of the amount of fever, has not been successful in abolishing it, and has presumably left other (and possibly unsuspected) channels of diffusion in their former patency. Taking the county of London alone, the return under consideration shows that 449 cases of typhoid or enteric fever were notified during the quarter, and that 74 of them terminated fatally. The number of cases ranged from one in the borough of Holborn to 36 in the borough of Stoke Newington, the populations of the two boroughs being not very dissimilar. Of the 30 boroughs of the county (including the Port) not one escaped. Nowhere was there freedom, and nowhere was there any great prevalence. If these cases had been occasioned by polluted water it is difficult to believe that the pollution would not, in some of the localities concerned, have exerted a much more extended influence, or even led to the occurrence of something which might be described as an epidemic.

